# Effect of Uric Acid-Lowering Agents on Patients With Heart Failure: A Systematic Review and Meta-Analysis of Randomised Controlled Trials

**DOI:** 10.3389/fcvm.2021.639392

**Published:** 2021-05-11

**Authors:** Hongxuan Xu, Yunqing Liu, Lingbing Meng, Li Wang, Deping Liu

**Affiliations:** ^1^Department of Cardiology, Beijing Hospital, National Center of Gerontology, Institute of Geriatric Medicine, Chinese Academy of Medical Sciences, Beijing, China; ^2^The Key Laboratory of Geriatrics, Beijing Institute of Geriatrics, Beijing Hospital National Center of Gerontology, National Health Commission, Institute of Geriatric Medicine, Chinese Academy of Medical Sciences, Beijing, China; ^3^Peking Union Medical College, Chinese Academy of Medical Science, Beijing, China; ^4^Departments of Neurology, Beijing Hospital, National Center of Gerontology, Institute of Geriatric Medicine, Chinese Academy of Medical Sciences, Beijing, China; ^5^Peking University Health Science Centre, Peking University Fifth School of Clinical Medicine, Beijing, China

**Keywords:** uric acid, hyperuricemia (HUA), heart failure, left ventricular ejection fraction, six minute walk test, B type natriuretic peptide, mortality

## Abstract

**Background:** Elevated serum uric acid (SUA) level is considered an independent predictor of all-cause mortality and the combined endpoint of death or readmission in cardiovascular disease patients. However, the causal relationship between uric acid-lowering therapies (ULTs) and heart failure is still controversial.

**Design:** Meta-analyses were performed to systematically compile available evidence to determine the overall effect of ULTs on heart failure patients.

**Method:** We conducted this systematic review following the PRISMA statement guidelines. Databases were searched to identify randomised controlled trials related to the influence of a ULT intervention in people with heart failure. Data extracted from the included studies were subjected to a meta-analysis to compare the effects of ULTs to a control.

**Results:** Pooled analysis of left ventricular ejection fraction (LEVF) showed an insignificant result towards the ULT group (MD, 1.63%; 95%CI, −1.61 to 4.88; *p* = 0.32; three studies). Pooled analysis of the 6-Minute Walk Test (6MWT) showed an insignificant result towards the ULT group (MD, 4.59; 95%CI, −12.683 to 22.00; *p* = 0.61; four studies). Pooled analysis of BNP/NT-pro-BNP led to a nearly statistically significant result towards the ULT group (SMD, −0.30; 95%CI, −0.64 to 0.04; *p* = 0.08; five studies). Pooled analysis of all-cause mortality and cardiovascular death between ULTs (all XOIs) and placebo did not show a significant difference (RR, 1.26; 95% CI, 0.74 to 2.15, *p* = 0.39).

**Conclusion:** ULTs did not improve LVEF, BNP/NT-pro-BNP, 6MWT, all-cause mortality, and CV death in heart failure patients. UA may just be a risk marker of heart failure.

## Introduction

Since we lack the uricase enzyme, which oxidises uric acid (UA) to allantoin, UA is the final metabolite of purine's degradation by xanthine oxidase (XO) enzyme in humans and some primates. The liver, intestines, muscle, and vascular endothelium produce UA from food digestion and endogenously synthesised purine compounds. Hyperuricemia is defined as having a serum uric acid (SUA) level of >7.0 mg/dl in men and >5.7 mg/dl in women, and the prevalence of hyperuricemia in the USA is above 20% both in men and women (1).

Heart failure (HF) is a lethal chronic condition that affects at least 26 million people worldwide and increases prevalence (2). Despite HF treatments having progressed remarkedly over the past three decades, HF with preserved ejection fraction (HFpEF), where its morbidity and mortality are on par with HF with reduced ejection fraction (HFrEF), still needs treatments proven to be effective (3).

UA has an antioxidant effect that is considered preventive for cardiovascular disease (CVD), such as atherosclerosis (4, 5). Nevertheless, xanthine oxidoreductase (XOR) produces UA and brings out reactive oxygen species (ROS); inhibiting XO activity improves myocardial function in patients with idiopathic dilated cardiomyopathy, suggesting that XO activity may contribute to energy metabolism in human heart failure (6, 7). These findings implicate an intricate role of xanthine oxidase activity involved in the development of CVD.

A meta-analysis involving 12,854 acute heart failure patients indicated that elevated SUA level is an independent predictor of all-cause mortality and the combined endpoint of death or readmission in acute heart failure patients (8). One cohort study showed that UA is a predictor of both short-term and long-term mortality of the elderly admitted to the medical department (9, 10). Although a plethora of evidence supports the association between hyperuricemia and increased mortality risk, it is still controversial whether UA plays a role as a risk marker or risk factor in CVD and cardiometabolic disease.

Synthesised RCT data showed no difference in cardiovascular events among urate-lowering therapies (ULTs) in gout (11). Xanthine oxidase inhibitor (XOI) did not significantly reduce the risk of major adverse cardiovascular events and death (12). Conflicting evidence suggests a reverse causality of the relationship between SUA elevation and CVD (13). The purpose of this meta-analysis is to assess the effect of ULTs on HF patients.

## Methods

We conducted this systematic review under the Preferred Reporting Items for Systematic Reviews and Meta-Analysis (PRISMA) statement guidelines (14).

We carried out a keyword search using the terms “uric acid,” “heart failure,” and “randomised controlled trials” and ULTs like “Uricosuric” and “Xanthine oxidase inhibitor.” We searched Ovid MEDLINE, Web of Science, EMBASE, PubMed, ClinicalTrials, Google Scholar, and Cochrane Library databases from the start date of September 20, 2020. These databases were searched using a combination of subject headings (such as Medical Subject Headings), filters (such as “RCT”), or PICOS framework when available. We also reviewed the references of included studies to identify additional pertinent studies. We imposed no language or time restriction. A protocol was developed before commencing this review on PROSPERO [CRD42020209883].

### Inclusion and Exclusion Criteria

Two reviewers independently assessed the records identified from the search for eligibility. Any discrepancies were resolved by consensus. We included any randomised controlled trials comparing ULTs vs. placebo in adult heart failure patients. The target population was adults (aged 18 years and above) with heart failure (as defined by New York Heart Association). The outcome had to objectively measure cardiac capacity and mortalities. We excluded studies that were not placebo controlled and missing critical data. Studies that followed or treated the patients with XOI for <4 weeks or did not measure heart functions and mortalities were excluded. Studies whose intervention groups received co-interventions were also excluded.

### Study Quality

The two authors assessed study quality based on the seven domains defined by the Cochrane Collaboration tool for assessing the risk of bias (15): (1) random sequence generation; (2) allocation concealment; (3) blinding of participants and personnel; (4) blinding of outcome assessment; (5) incomplete outcome data; (6) selective reporting; and (7) other biases, including baseline imbalance, early stopping, and bias due to vested financial interest or academic bias.

### Data Extraction

One author (XHX) extracted all data, and both authors (XHX and LYQ) reviewed the data for accuracy. The following data were collected: country, duration of the trial, date of publication; numbers of individuals included, inclusion criteria, exclusion criteria, baseline characteristics, intervention, and control; outcomes: SUA change, left ventricular ejection fraction (LVEF), Six-Minute Walk Test (6MWT), brain natriuretic peptide/N-terminal Brain Natriuretic Peptide (BNP/NT-BNP), all-cause mortality, and cardiovascular death.

We used Origin 2019b (OriginLab Corporation) to extract data from the figures. We used the methods derived from Wan et al. to estimate the sample mean and standard deviation (SD) from the sample size, median, and interquartile range (16).

### Data Synthesis

#### Meta-Analyses

Meta-analysis was performed with Review Manager 5.4.1. We used a random-effect model and calculated the mean difference (MD) to generate pooled estimates of LVEF and 6MWT changes, a random-effect model and standard mean difference (SMD) to calculate the intervention effects of SUA and BNP/NT-BNP across studies. Mortality data were performed by risk ratio (RR) using random-effect Mantel-Haenszel methods (17). We calculated the standard deviation using an assumption of a 0.5 correlation or deriving correlation from studies with existing data for studies that did not report the standard deviation of the mean of change, following the Cochrane Handbook for Systematic Reviews of Interventions. The *I*^2^ statistic was used to assess the degree of statistical heterogeneity (0–40%: might not be important, 30–60%: moderate heterogeneity, 50–90%: substantial heterogeneity, 75–100%: considerable heterogeneity) (18). *P*-values <0.05 were considered statistically significant.

We conducted subgroup analyses to explore the potential causes of heterogeneity for treatment effect on different ULT methods (XOIs vs. uricosuric drugs).

#### Trial Sequential Analysis

Trial sequential analysis is a methodology that considers how much information is needed to anticipate a specific required information size (19). We used the TSA program version 0.9.5.10 Beta (Copenhagen Trial Unit) to adjust the confidence intervals due to sparse data and repetitive testing of cumulative data and calculate the required information size. If the cumulative Z-curve crosses a trial sequential monitoring boundary or enters the futility area, the required information size may have reached a sufficient level of evidence. Conversely, the conclusive evidence is insufficient if the Z-curve does not cross any boundaries. The required information size was calculated based on autogenerated empirical data per input data. We performed the trial sequential analysis at the level of an overall 5% risk of type I error and a power of 20%.

### Sensitivity Analysis

We conducted a *post-hoc* sensitivity analysis to assess a single study's impact on overall heterogeneity.

## Results

### Included Studies

**Figure 1:**
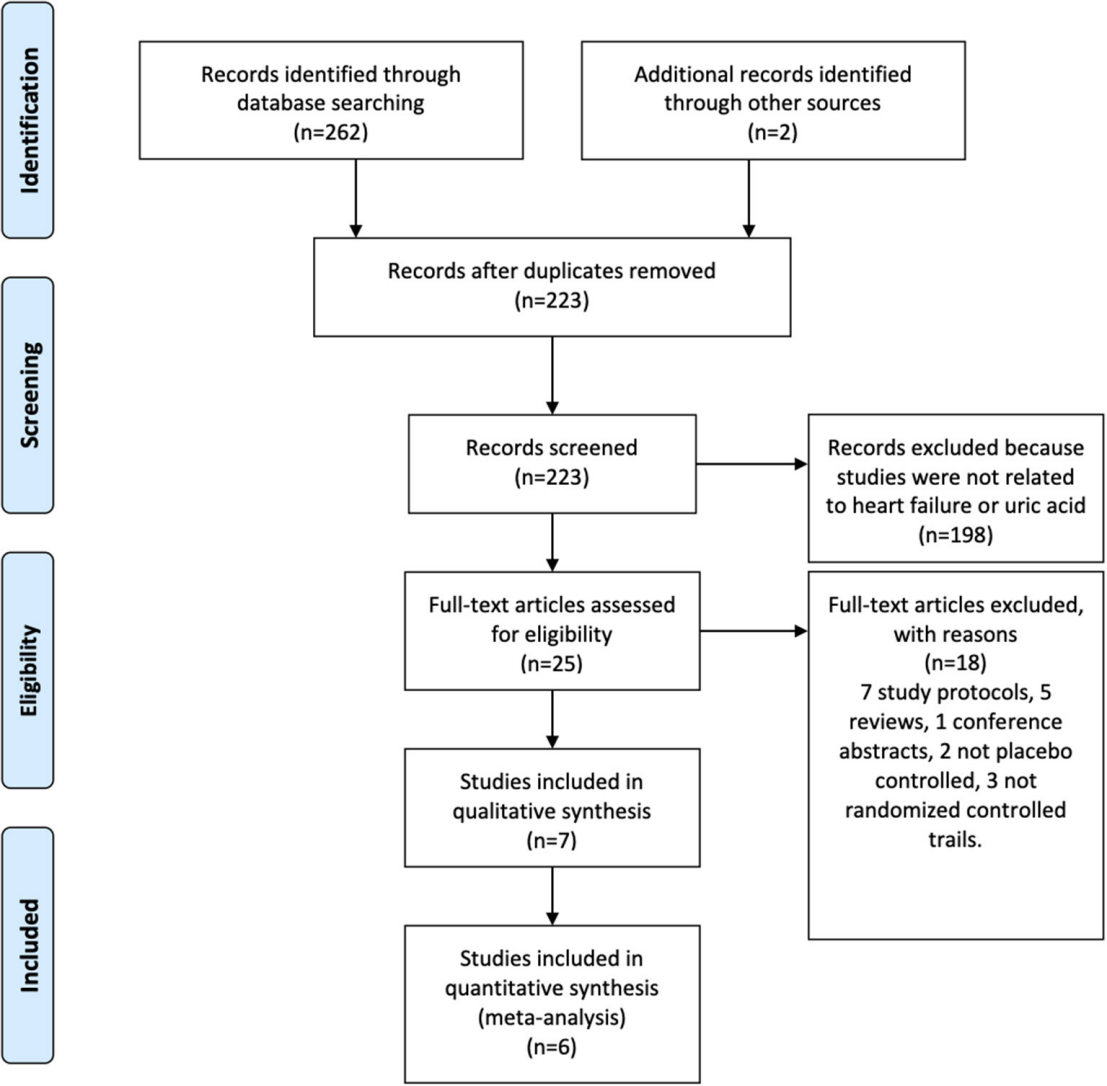
Preferred Reporting Items for Systematic Reviews and Meta-Analyses (PRISMA) flow diagram.

Six studies (20–25), with a total of 864 participants, fulfilled the inclusion criterion (Figure 1). SUA was assessed in five studies, LVEF in three studies, BNP/NT-BNP in five studies, 6MWT in four studies, and mortality in two studies. Table 1 summarises the characteristics of the included studies.

**Table 1:** Characteristics of included studies.

**References**	**Age (control/intervention)**	**Male %**	**Inclusion criteria**	**Intervention**	**Control**	**Duration**	**Intervention protocol**	**Outcomes measured**
Gavin and Struthers (20)	67	77	NYHA II-III	Allopurinol (*n* = 44)	Placebo (*n* = 44)	12 week	Allopurinol 300 mg/day	BNP, 6MWT
Cingolani et al. (21)	66.7/70.6	60/63	NYHA II-III, LVEF ≤40%, 6MWT <425 m	Oxypurinol (*n* = 21)	Placebo (*n* = 26)	1 month	Oxypurinol 100 mg/day a week, then 600 mg/day	SUA, LVEF, 6MWT
Hare et al. (22)	65/64	70/76	NYHA III-IV, LVEF ≤40%	Oxypurinol (*n* = 203)	Placebo (*n* = 202)	24-week	Oxypurinol 100 mg/day a week, then 600 mg/day	SUA, BNP, mortality
Ogino et al. (23)	59/62	75/74	NYHA I-III, hyperuricemia (UA >7.0 mg/dl)	Benzbromarone (*n* = 14)	Placebo (*n* = 14)	8-week	Benzbromarone 50 mg/day	SUA, BNP, LVEF
Givertz et al. (24)	63/63	78/86	LVEF ≤40%, SUA ≥9.5 mg/dl	Allopurinol (*n* = 128)	Placebo (*n* = 125)	24-week	Allopurinol 300 mg/day, then 600 mg/day if tolerated	SUA, NT-pro-BNP, LVEF, 6MWT, mortality
Robbins et al. (25)	57.7	80	NYHA II-IV, LVEF ≤40%	Probenecid (*n* = 20)	Placebo (*n* = 20)	4-week	Probenecid 2 g/d (twice daily)	NT-pro-BNP, 6MWT

*NYHA, New York Heart Association; SUA, serum uric acid; BNP, B-type natriuretic peptide; NT-pro-BNP, N-terminal-pro-B-type natriuretic peptide; LVEF, left ventricular ejection fraction; 6MWT, Six-Minute Walk Test*.

The most common SUA-lowering therapy was XOI (four out of six studies). The follow-up duration ranged from 4 to 24 weeks, with a median of 10 weeks. The mean age was 62 years (range, 58–69). Men accounted for 66% (range, 36–82%) of the pooled population. The ULTs were uricosuric drugs (benzbromarone, probenecid) and XOIs (allopurinol, oxypurinol).

### Risk of Bias and Grade

**Figure 2:**
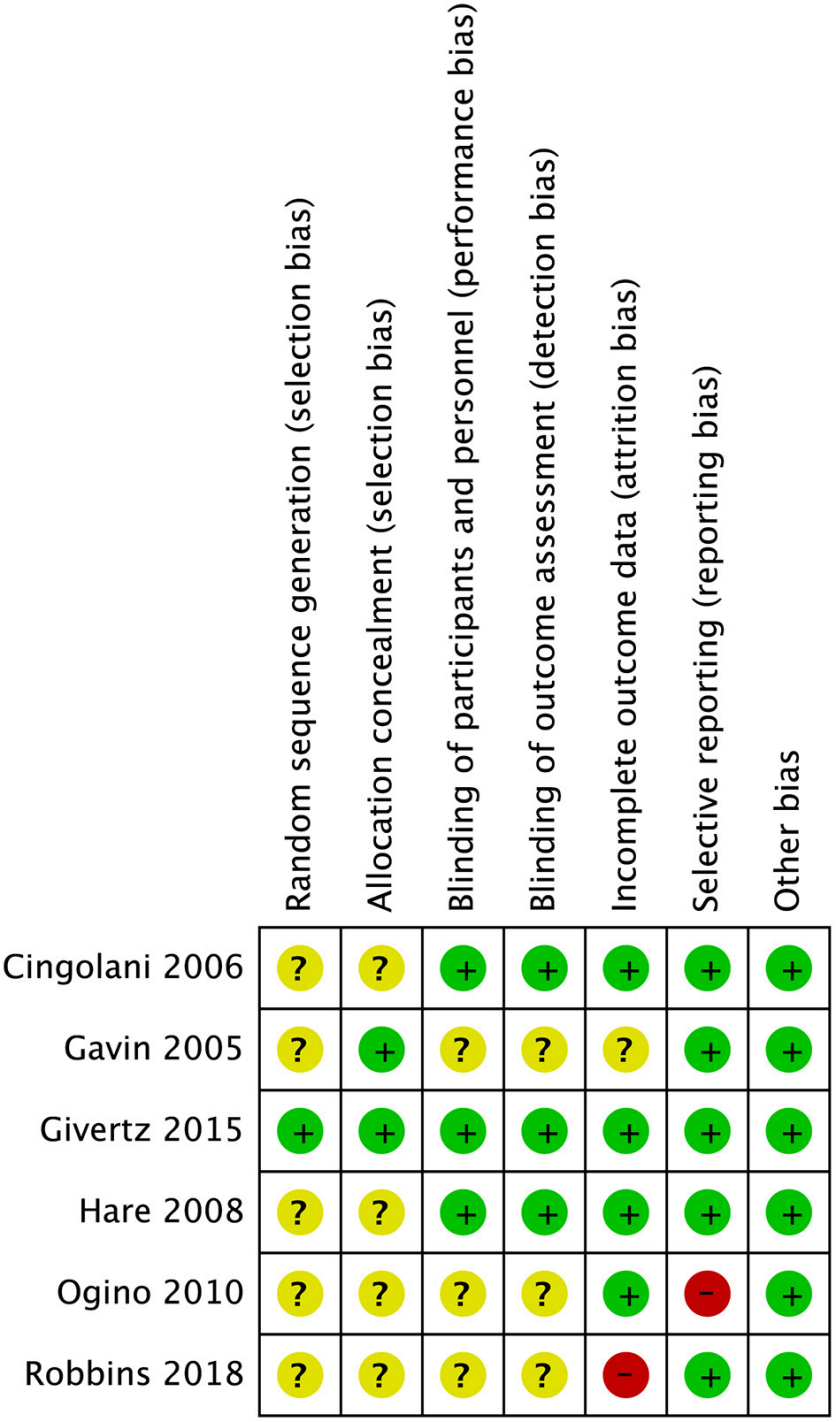
Risk of bias summary.

Five studies did not provide enough details regarding the method of randomisation other than just stating that it was a randomised trial (20–23, 25). Hence, the random sequence generation and allocation concealment were unclear. One study was deemed to have a high risk of incomplete outcome data (25). Figure 2 summarised the risk of bias. Table 2 summarised the GRADE evidence table.

**Table 2:** GRADE evidence table.

**Certainty assessment**							**No of patients**		**Effect**		**Certainty**	**Importance**
**No of studies**	**Study design**	**Risk of bias**	**Inconsistency**	**Indirectness**	**Imprecision**	**Other considerations**	**Uric lowering therapies**	**Placebo**	**Relative (95% CI)**	**Absolute (95% CI)**		
**Left ventricular ejection fraction**												
3	Randomised trials	Not serious	Not serious	Not serious	Serious^a^	None	163	165	–	MD **1.63 % higher** (1.61 lower to 4.88 higher)	⊕⊕⊕○ moderate	Important
**B-type natriuretic peptide and N-terminal-pro-B-type natriuretic peptide**												
5	Randomised trials	Not serious	Serious^b^	Not serious	Not serious	None	387	383	–	SMD **0.3 SD lower** (0.64 lower to 0.04 higher)	⊕⊕⊕○ moderate	Important
**Six-Minute Walk Test**												
4	Randomised trials	Not serious	Not serious	Not serious	Not serious	None	170	167	–	MD **3.47 m lower** (14.84 lower to 7.9 higher)	⊕⊕⊕⊕ high	Critical
**Cardiovascular death**												
2	Randomised trials	Not serious	Not serious	Not serious	Not serious	None	13/331 (3.9%)	11/327 (3.4%)	**RR 1.16** (0.41 to 3.23)	**5 more per 1,000** (from 20 fewer to 75 more)	⊕⊕⊕⊕ high	Critical
**All-cause mortality**												
2	Randomised trials	Not serious	Not serious	Not serious	Not serious	None	18/331 (5.4%)	13/327 (4.0%)	**RR 1.36** (0.68 to 2.73)	**14 more per 1,000** (from 13 fewer to 69 more)	⊕⊕⊕⊕ high	Critical

a *The numbers of studies and participants are relatively low*.

b *The results of the two subgroups are inconsistent. RR: relative risk. m: meters*.

### Left Ventricular Ejection Fraction

**Figure 3:**
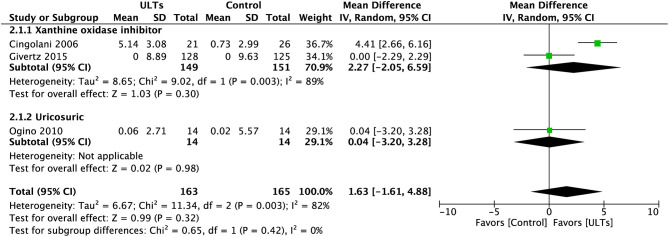
Meta-analysis of left ventricular ejection fraction.

The LVEF at baseline in the control group (23.4% ± 7.7) was significantly lower than the intervention group (25.6% ± 8.6) in (24). The LVEF at baseline in Ogino et al. (49.5 ± 12.7) is higher than the other two studies due to different inclusion criteria. Pooled analysis of LEVF (Figure 3) showed an insignificant result towards the ULT group (MD, 1.63%; 95%CI, −1.61 to 4.88; *p* = 0.32; three studies) with substantial heterogeneity (*I*^2^ = 82%), a test for subgroup heterogeneity showed low heterogeneity (*I*^2^ = 0%).

**Figure 4:**
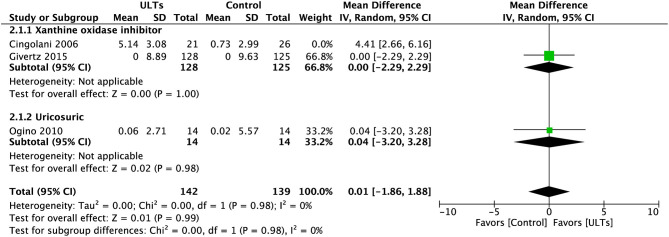
Meta-analysis of left ventricular ejection fraction without Cingolani et al.

The effect size was mainly powered by Cingolani et al. (21). After excluding this study, the result was still insignificant (MD, 0.01; 95% CI, −1.86 to 1.88; *p* = 0.99) but with low heterogeneity (*I*^2^ = 0%). A test for subgroup heterogeneity showed low heterogeneity (*I*^2^ = 0%) (Figure 4).

**Figure 5:**
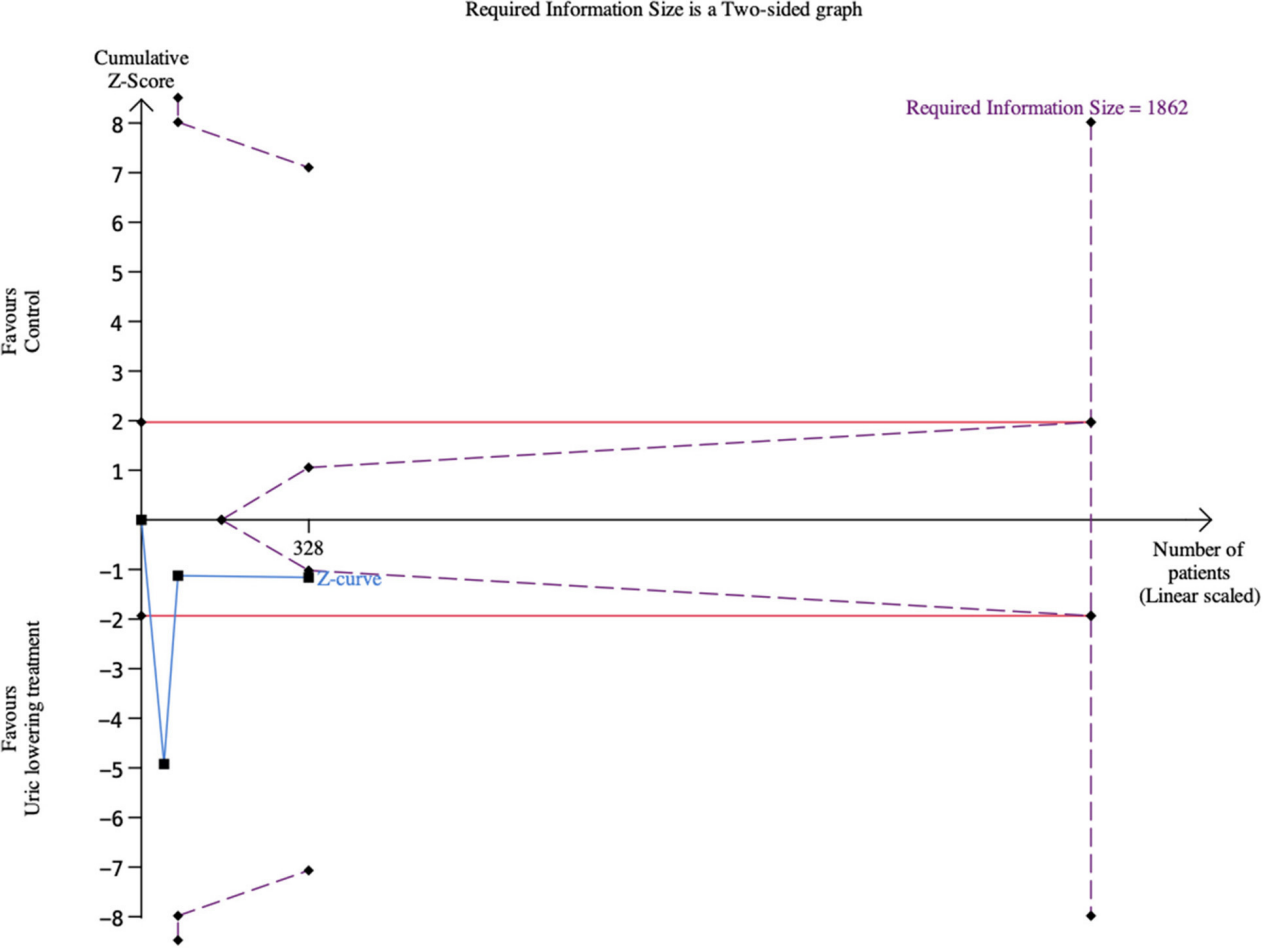
Trial sequential analysis of left ventricular ejection fraction.

TSA using a random-effect model showed that the cumulative Z-curve of LVEF neither crossed a trial sequential monitoring boundary nor entering the futility area (Figure 5).

### B-Type Natriuretic Peptide and N-Terminal-Pro-B-Type Natriuretic Peptide

**Figure 6:**
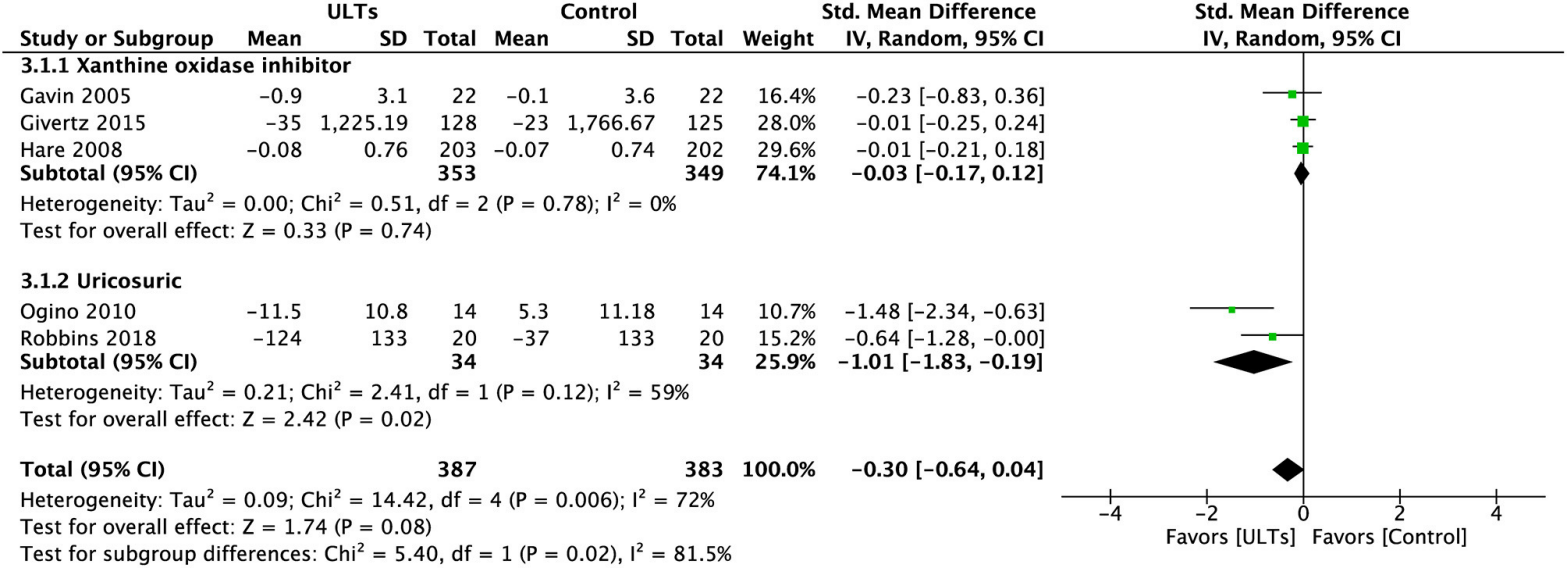
Meta-analysis of B-type natriuretic peptide and N-terminal-pro-B-type natriuretic peptide.

Pooled analysis of BNP/NT-pro-BNP (Figure 6) showed a nearly statistically significant result towards the ULT group (SMD, −0.30; 95% CI, −0.64 to 0.04; *p* = 0.08; five studies) with substantial heterogeneity (*I*^2^ = 72%), a test for subgroup heterogeneity showed substantial heterogeneity (*I*^2^ = 81.5%).

The difference between subgroups was significant (*p* = 0.02). The XOI group showed an insignificant result (SMD, −0.03; 95% CI, −0.17 to 0.12; *p* = 0.78; three studies) with low heterogeneity (*I*^2^ = 0%). The uricosuric group led to a significant effect (SMD, −1.01; 95% CI, −1.83 to 0.19; *p* = 0.02; two studies) with moderate to substantial heterogeneity (*I*^2^ = 59%).

### Six-Minute Walk Test

**Figure 7:**
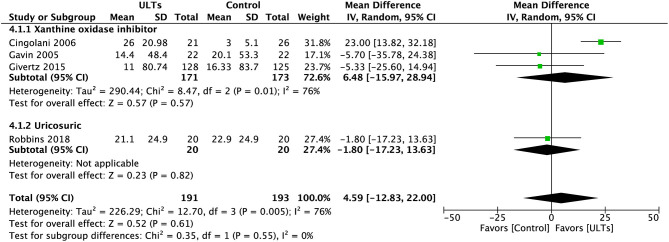
Meta-analysis of 6-Min Walk Test.

Pooled analysis of 6MWT (Figure 7) showed an insignificant result towards the ULT group (MD, 4.59; 95% CI, −12.683 to 22.00; *p* = 0.61; four studies) with substantial heterogeneity (*I*^2^ = 76%), a test for subgroup heterogeneity showed low heterogeneity (*I*^2^ = 0%).

**Figure 8:**
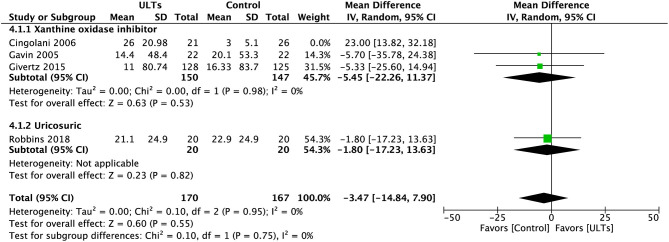
Meta-analysis of 6-Min Walk Test without Cingolani et al.

However, similar to LVEF, after excluding Cingolani et al., the overall heterogeneity drastically reduced to *I*^2^ = 0%. The effect size turned towards the control group (MD, −3.47; 95% CI, −14.84 to 7.90; *p* = 0.55; three studies) (Figure 8).

**Figure 9:**
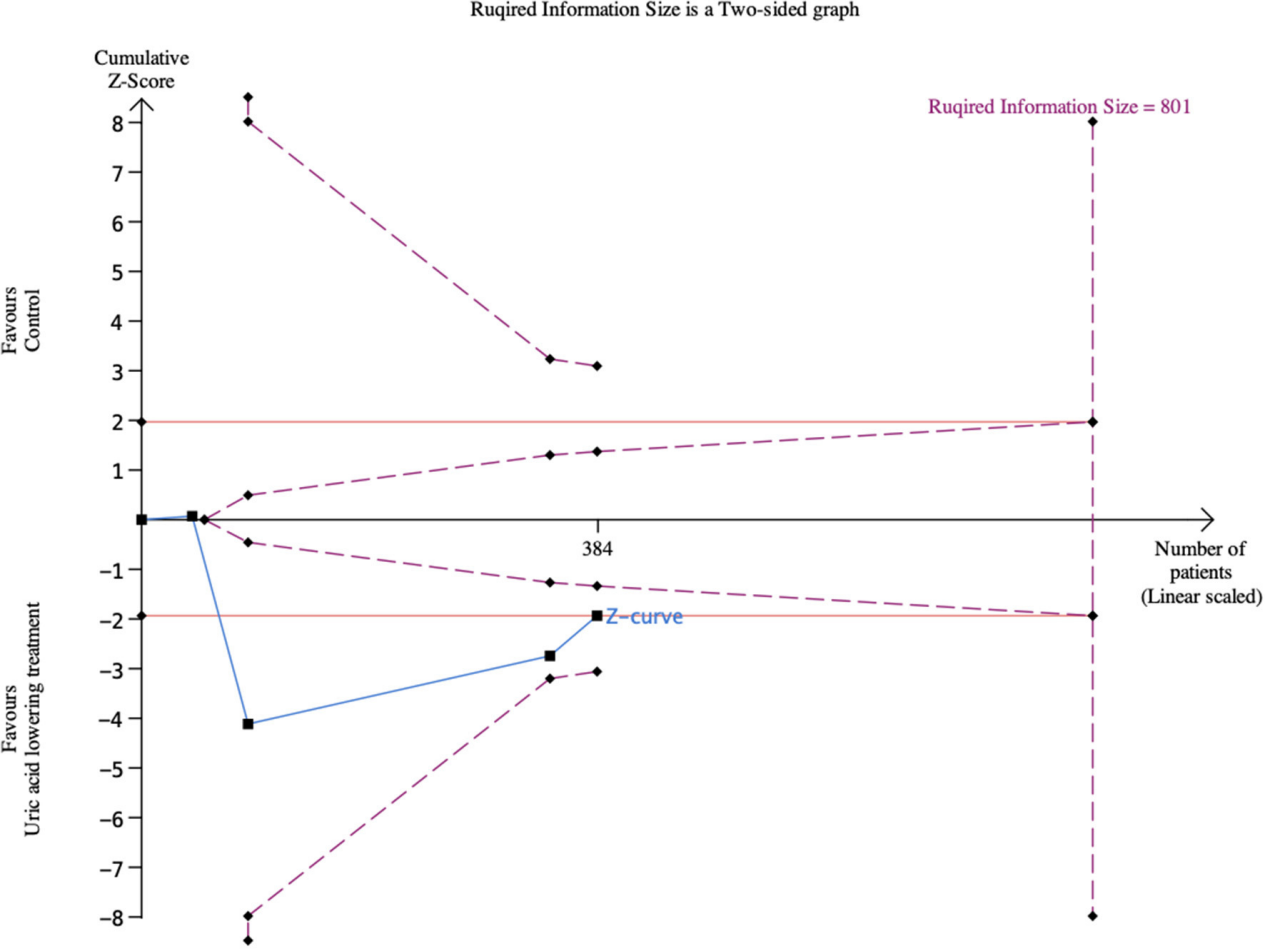
Trial sequential analysis of 6-Min Walk Test.

TSA using a random-effect model showed that the cumulative Z-curve of 6MWT neither crossed a trial sequential monitoring boundary nor entering the futility area (Figure 9).

### All-Cause Mortality and Cardiovascular Death

**Figure 10:**
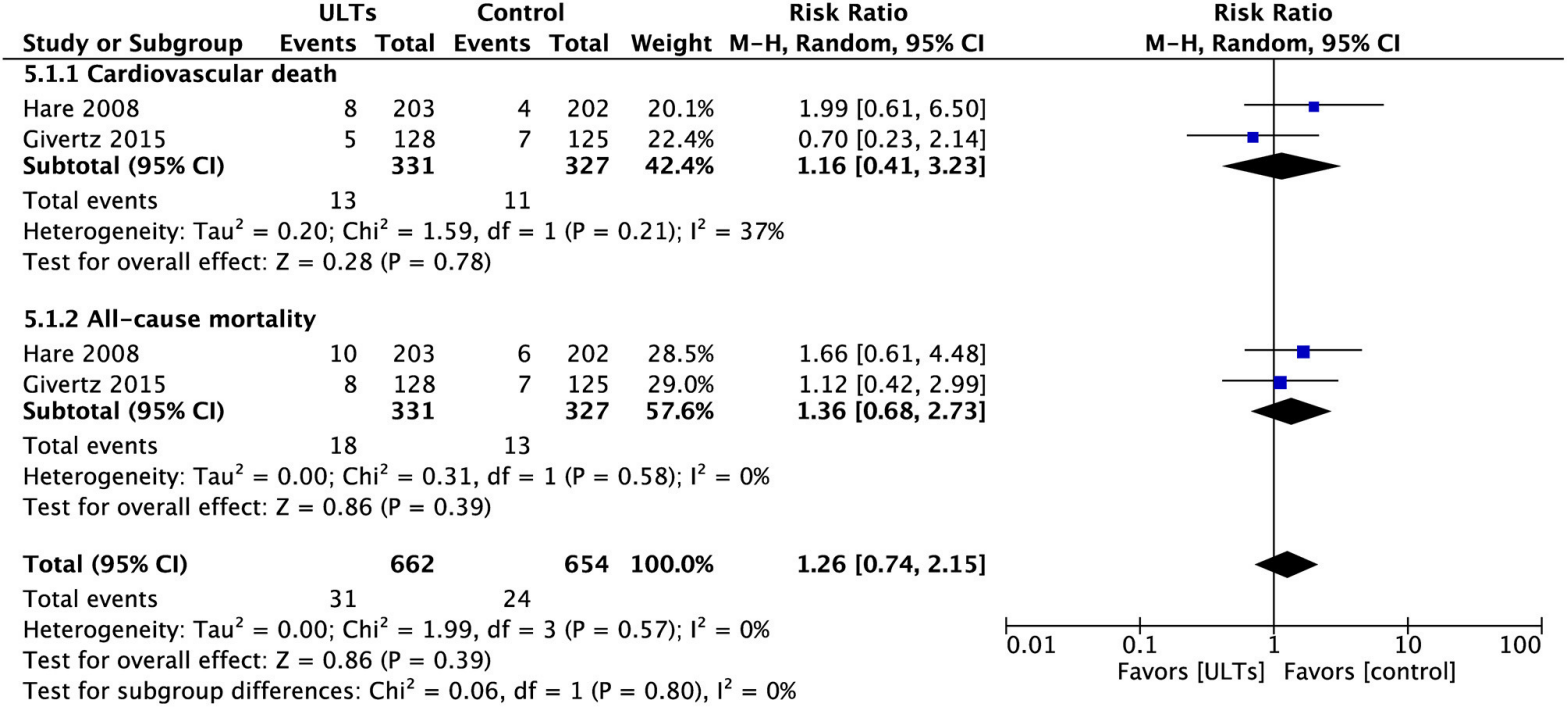
Meta-analysis of all-cause mortality and cardiovascular death.

Overall, the pooled analysis of all-cause mortality and cardiovascular death between ULTs (all XOIs) and placebo did not show a significant difference (RR, 1.26; 95% CI, 0.74 to 2.15; *p* = 0.39) with low heterogeneity (*I*^2^ = 0%) (Figure 10).

The all-cause mortality is slightly in favour of the control group (RR, 1.36; 95% CI, 0.68 to 2.73; *p* = 0.39).

## Discussion

This meta-analysis examined the relationship between ULTs and all-cause mortality, CV mortality, BNP/NT-pro-BNP, 6MWT, and LVEF in HF patients. We did not find any statistically significant difference between ULTs and placebos. However, it did show a significant effect in favour of the uricosuric group (benzbromarone and probenecid) on BNP/NT-pro-BNP.

A large quantity of experimental and clinical data suggests that oxidative stress contributes to ventricular and vascular remodelling and disease progression in HF. XO is a potent source of oxidative stress, and therefore an intuitive target for therapy.

Probenecid is an agonist of transient receptor potential vanilloid 2 (TRPV2), which may increase myocardial contractility *via* increased calcium cycling on a beat-to-beat basis (26). It increased the force generation and calcium sensitivity of single cardiomyocytes, consistent with improved cardiac function observed in patients with HFrEF (25). Even though the results of Robbins et al. were compatible with most pooled results of included studies, it was a source of underlying heterogeneity. Cingolani et al. (21) had the most significant SUA reduction and the shortest follow-up period; it may indicate a short-term protective effect of XOI.

Previous studies indicated that XOI improves peripheral vasodilator capacity and blood flow, both locally and systemically in hyperuricemia CHF patients (27). Moreover, acute exposure of arteries and aortas isolated from aged WKY rats to a high UA concentration did not provoke changes in endothelial function (28). Allopurinol may improve endothelial function by reducing vascular oxidative stress and not in urate reduction (29). However, similar to our results, several recent meta-analyses, including observational studies, demonstrated that XOI did not have a long-term protective effect regarding all-cause mortality and cardiovascular death (30, 31).

Imbalanced nitric oxide (NO) bioavailability may induce impairment of endothelium-dependent relaxation due to an imbalance between endothelium-derived relaxing factors (EDRFs) and endothelium-derived contracting factors (EDCFs) (32). XOR and UA may decrease endothelial NO bioavailability through multiple mechanisms (33). Despite that ROS is produced during the XOR process, XOR is not the only pathway that contributes to nitroso-redox imbalance. Other enzymes also have ROS, such as NADPH oxidase enzymes, the respiratory chain in the mitochondria, and superoxide dismutase. XOI might be inadequate to curtail the cascade of ROS accumulated in HF patients.

Along with these pathophysiological assumptions, researchers observed that elevated UA levels are related to HF markers, such as measures of myocardial mechanical and energetic efficiency, LVEF, LV stroke volume, cardiac output, cardiac remodelling, endothelium dysfunction, plasma BNP levels, and CHF disease progression (27). It was also considered an independent predictor of mortality in HF patients and a robust dose-dependent increase of risk parallel to higher UA levels. Several studies showed a U-shaped association between UA and all-cause or CV mortality; SUA ≥8 or <4 mg/dl independently predicts higher all-cause and CVD-related mortality in the elderly (34, 35). Accordingly, we pooled several studies with an HF population with reduced ejection fraction and with or without elevated serum UA levels. In the OPT-CHF and EXAT-HF, both trials failed to show a beneficial effect regarding mortality. Notably, OPT-CHF included patients regardless of the UA level at baseline were included, while EXAT-HF only included patients with elevated serum uric acid levels (UA ≥9.5 mg/dl). However, the consistency of included studies was high despite the different inclusion criteria. We found that using ULTs, including XOI and uricosuric drugs on HF patients with or without hyperuricemia, did not improve heart function, exercise capacity, all-cause, and CV mortality. Therefore, UA may not be a causative risk factor in HF patients.

## Limitations

Even though most studies in this meta-analysis included patients with HFrEF and it is the most common type of HF (36), two studies (21, 23) did not specifically include patients with HFrEF. In HFpEF, the endothelial cells that line small blood vessels in the heart become dysfunctional and fail to synthesise adequate amounts of NO (37). Evidence showed that XOI could decrease NO production (38).

Since there is only one ongoing study (39), we cannot include the XO selective, uric acid-lowering drug febuxostat. However, febuxostat recently showed to increase the risk of future development of cardiovascular disease (40).

The overall sample size was small, and even though we included only randomised, placebo-controlled trials, different patient inclusion, exclusion criteria, and dosing protocol can lead to potential heterogeneity. The duration of included studies was also various. Therefore, the conclusions may be influenced by publication bias and should be regarded as preliminary.

## Conclusion

ULTs did not improve LVEF, BNP/NT-pro-BNP, 6MWT, all-cause mortality, and CV death in HF patients. UA may just be a risk marker of HF.

## Data Availability Statement

The original contributions presented in the study are included in the article/supplementary material, further inquiries can be directed to the corresponding author/s.

## Author Contributions

HX and DL contributed to the study's conception. HX and YL screened studies and extracted data. HX performed the data analyses and wrote the manuscript. DL, LM, LW, and YL helped perform the analysis with constructive discussions. All authors had read and approved the final manuscript.

## Conflict of Interest

The authors declare that the research was conducted in the absence of any commercial or financial relationships that could be construed as a potential conflict of interest.
